# Increasing trend of diabetes combined with hypertension or hypercholesterolemia: NHANES data analysis 1999–2012

**DOI:** 10.1038/srep36093

**Published:** 2016-11-02

**Authors:** Yongfeng Song, Xiaoyun Liu, Xiaolin Zhu, Bin Zhao, Bo Hu, Xia Sheng, Lan Chen, Miao Yu, Tao Yang, Jiajun Zhao

**Affiliations:** 1 Department of Endocrinology and Metabolism, Shandong Provincial Hospital affiliated to Shandong University, No. 324 Jingwu Weiqi Road, Jinan 250021, China; 2 Department of Endocrinology, The First Affiliated Hospital of Nanjing Medical University, No. 300 Guangzhou Road, Nanjing 210029, China; 3 MSD China Holding Company, No. 1601 Nanjing West Road, Shanghai 200040, China; 4 Department of Quantitative Health Sciences, Cleveland Clinic. No. 9500 Euclid Avenue Cleveland, Ohio 44195, USA; 5 Department of Endocrinology, Key Laboratory of Endocrinology, Ministry of Health, Peking Union Medical College Hospital, Diabetes Research Center of Chinese Academy of Medical Sciences & Peking Union Medical College, No. 1 Shuaifu Garden, Beijing 100730, China

## Abstract

In order to prevent cardiovascular endpoints, control of diabetes, hypertension and hypercholesterolemia is a necessity as those risk factors frequently occur in combination. Prevalence trends of concurrent diabetes, hypertension and hypercholesterolemia in 36,673 subjects were obtained from the National Health and Nutrition Examination Survey (NHANES) from 1999–2012. The prevalence of concurrent diabetes, hypertension and hypercholesterolemia increased from 3% in 1999–2000 to 6.3% in 2011–2012 (*P* < 0.001). The diabetes with concurrent hypertension or hypercholesterolemia incidences also increased significantly, while the occurrence of concurrent hypertension and hypercholesterolemia was stable over the study period. Overall medical drug treatments for concurrent diabetes, hypertension, hypercholesterolemia were improved from 69.8% in 1999–2006, to 82.4% in 2007–2012 (*P* = 0.002). Treatment cost coverage rates in any combinations with diabetes were 79–82.4% and 90.7% in the subgroup of concurrent hypertension and hypercholesterolemia. General treatment goal achievement rates were <25%, the lowest rate being 14.2% in the subject groups with three combined risk factors. The treatment goal achievement rates in two subgroups with concurrent diabetes were 20.1% (with hypertension) and 17% (with hypercholesterolemia) and 24.5% in the group without diabetes. Cost coverage improved in all combinations, but the general treatment goal achievement rates were low, especially in the groups with concurrent diabetes.

It is well documented that cardiovascular disease affects about two million American individuals each year and is the leading cause of mortality in the United States, making it a major medical challenge^1^.

The cardiovascular risks include hypertension, dyslipidemia, diabetes, obesity, smoking, age, etc. Three medical conditions namely diabetes, hypertension and hypercholesterolemia are recognized as major risk factors not only for cardiovascular disease but also for stroke and other conditions^2^,^3^,^4^,^5^,^6^,^7^. These three conditions present great challenges and constitute a heavy burden on health care in the United States and worldwide as well^8^,^9^,^10^.

During recent decades, there has been a substantial increase in the prevalence of diabetes along with obesity in adults in the United States. Although the control of diabetes has significantly improved, it still presents a major challenge^11^,^12^. The prevalence of hypertension was reported to be stable over time, while the treatment and control of hypertension has greatly improved^13^,^14^,^15^. However, the prevalence of hypercholesterolemia has remained at almost epidemic levels^16^,^17^,^18^. Furthermore, while significant improvements have been made in the therapeutic control of hypercholesterolemia, they are still less than satisfactory and the implementation of nationwide education programs to encouraging awareness of the severe risks associated with hypercholesterolemia have not been effective.

Although each of the three conditions produces unique cardiovascular risk factors, increasingly two or more are frequently found within one individual. For example, of hypertensive patients, 60% of them also present with diabetes, and 73% with dyslipidemia^19^. Of diabetic patients, 30% of them may also have dyslipidemia^20^. Data from the National Health Interview Survey (NHIS) found that approximately 26% of US adults had multiple (≥2) chronic conditions^21^. Any form of two or more of these conditions frequently coexisted^22^,^23^,^24^. Previous studies have indicated that the presence of multiple risk factors additively increases the risk of cardiovascular diseases^25^. While traditional disease management and epidemiology studies have focused on individual disease, there is robust evidence that concurrent comorbidities, especially the presence of any form of combination of diabetes, hypertension and dyslipidemia, has an even higher risk for the development of cardiovascular and renal diseases than each condition alone. Their impact on CVD events is thought to be additive^26^. Robust evidence supports the integrated management and measurement of cost effective risk factors, especially abnormalities in blood glucose, blood pressure and blood lipids^27^,^28^,^29^.

It is of great clinical interest for physicians and clinicians to study concurrent diabetes, hypertension and hypercholesterolemia in patients as it represents a unique clinical panorama, with important consequences for the patients. For concurrent diabetes and hypertension, the optimal selection of anti-hypertensive drugs and the optimal target are different from a patient suffering solely from hypertension, as recommended by the American Diabetes Association (ADA)^30^,^31^. The same also applies to concurrent diabetes and hypercholesterolemia. For treatment of concurrent hypercholesterolemia and diabetes, the target is more strict compared to hypercholesterolemia without diabetes, as recommended by the American College of Cardiology (ACA)/American Heart Association (AHA)^32^,^33^. For concurrent hypertension and hypercholesterolemia, this subgroup of patients would manifest itself with higher body mass indexes and being more prone to insulin resistance, compared to patients with hypertension alone^24^.

Unfortunately, there is a paucity of data available about the prevalence of concurrent comorbidities. There has been only one study concerning the prevalence of diabetes, hypertension and hypercholesterolemia in Switzerland; however, the authors reported the situation for each disease separately^34^. Two publications documented the prevalence of a combination of hypertension and dyslipidemia in the United Kingdom^35^ and the U.S.^36^, respectively, but they did not uncover a trend over time or the combined presence of diabetes.

The primary objectives of this investigation were to estimate the national trends in the prevalence, management and control of a combination of diabetes, hypertension and hypercholesterolemia in U.S. adults from 1999 to 2012. Combinations of any two conditions were also examined, as those groups might be of special interest to specialists.

## Methods

### Data

The National Health and Nutrition Examination Survey (NHANES) is a cross-sectional survey conducted by the National Center for Health Statistics, a branch of the Centers for Disease Control and Prevention (CDC). The study was approved by the National Center for Health Statistics Institutional Ethics Review Board, and all adult participants provided written informed consent^37^. All methods were performed in accordance with the Declaration of Helsinki regarding ethical standards for research involving human subjects. NHANES uses a complex, multistage and stratified sampling design to select a sample representative of the civilian and non-institutionalized resident population of the United States. The sampling procedure consists of four stages: primary sampling units (mostly counties), segments, households and individuals, respectively. Participants in NHANES filled in s at home, followed by physical and laboratory examinations at a mobile examination center. The NHANES questionnaires, laboratory tests and examinations have been previously described in the literature^38^.

NHANES interviewed and examined some 5,000 participants annually and the survey data released on a 2-year cycle. Response rates for participation in both interviews and physical examinations were similar across cycles and ranged from 75% to 80%^39^. The current investigation was based on NHANES data from 1999 to 2012, including all adult participants (aged ≥20 years), with complete data related to the definitions of diabetes, hypertension and hypercholesterolemia (*vide infra*). Pregnant women were excluded from the analysis.

### Definitions

*Diabetes* was defined as a self-reported diagnosis by the participants or hemoglobin A1c (HbA1c) ≥6.5% or both. Self-reported diabetes was defined as the participant answered yes to at least one of the survey questions “Doctor said you have diabetes”, “now taking insulin” and “now taking diabetic pills to lower your blood sugar?” 25.6% of the diabetes cases were self-reported. HbA1c was measured in whole blood samples using high-performance liquid chromatography, performed on instruments certified by the National Glycohemoglobin Standardization Program and standardized to the reference method used in the Diabetes Control and Complications Trial^11^. We did not distinguish between type 1 and 2 diabetes.

*Hypertension* was defined as a systolic blood pressure (SBP) ≥140 mmHg or a diastolic blood pressure (DBP) ≥90 mmHg, or patients being treated with antihypertensive medication. The use of antihypertensive medication was defined as the participant answered yes to the survey question (Are you now taking prescribed medicine for HBP?). 38.8% of the hypertension cases were self-reported. Systolic and diastolic blood pressure levels were measured three to four times by mercury sphygmomanometer using a standard protocol to reduce variability. SBP and DBP were calculated by averaging multiple measurements.

Although low-density lipoprotein cholesterol (LDL) has been widely used as the standard biomarker for diagnosing lipid abnormalities, it was only measured in a subsample of the NHANES participants. To maximize the sample size, total serum cholesterol was used to define hypercholesterolemia. More specifically, *hypercholesterolemia* was defined as total serum cholesterol ≥200 mg/dL or on any concurrent pharmacologic lipid-lowering treatment or both. The use of therapy was based on an affirmative response to the survey question (Are you now following this advice to take prescribed medicine?). 17.0% of the hyperlipidemia cases were self-reported.

Treatment of diabetes was defined as a participant on insulin or other oral anti-diabetic drugs. Treatment of hypertension was defined as a patient taking antihypertensive medication. Treatment of hypercholesterolemia was defined as a patient taking prescribed lipid-lowering medication. The diabetes treatment goal achievement was defined as a HbA1c LEVEL <0.5%. The hypertension treatment goal achievement was defined as a SBP <140 mmHg and a DBP <90 mmHg, and the cholesterol treatment goal achievement was defined as total cholesterol <200 mg/dL.

The presence of combined conditions was defined as a participant who had multiple conditions at the same time. Treatment of combined conditions was defined as the participant receiving treatment for all their diagnosed conditions. Successful treatment of combined conditions was defined as all conditions under control.

Demographic and social-economic characteristics considered in the analyses included age, gender, race or ethnicity, body mass index (BMI), education level, income level and marital status. BMI was calculated from the measured height and weight and categorized into the following categories: <25, (25, 30) and ≥30 kg/m^2^. Race/ethnicity was self-reported as non-Hispanic white, non-Hispanic black, Mexican American, other Hispanic and non-Hispanic other. Education levels were classified into three categories: <high school, high school and >high school diploma. Income levels were based on the measure of income-to-poverty ratio and were classified into (0, 1.30), (1.30, 3.50) and >3.50 according to the Supplemental Nutrition Assistance Program (SNAP, formerly the Food Stamp Program) guidelines by the U.S. Department of Health and Human Service^40^. Married status was dichotomized into married/living with partner or other.

### Statistical Analysis

In general, participant characteristics were summarized as means ± standard deviations, medians and inter-quartile ranges or frequencies and percentages as appropriate on the 2-year survey cycle. The prevalence rate of each combined conditions was estimated per survey cycle. According to the NHANES Analytic and Reporting Guidelines, the estimation of the prevalence took into account selection probabilities, complex sample design and non-response and non-coverage by using appropriate sample weights to ensure unbiased estimation^41^,^42^. Standard errors associated with the prevalence estimates were obtained using Taylor series linearization. The trends of the prevalence over time were examined by using logistic regression models, which included the median year of the survey cycle as a continuous covariate. The models further controlled the participant’s characteristics. Subgroup analysis was performed for different demographic or social-economic strata by specifying the DOMAIN statement for the SURVEY procedure in SAS. The trends for each subgroup controlled for all other characteristics. The p values for trend were calculated based on testing null hypothesis that the slope of linear regression (prevalence was outcome and cycle year was a continuous predictor) was 0.

For management and control analyses, the denominator was the number of participants with corresponding concurrent conditions. Since these groups have much smaller sample sizes, we combined all 2-year survey cycles into two larger cohorts (1999–2006 and 2007–2012) for better estimation of the parameters for management and control. Statistical significance was established with a two-sided *P*-value < 0.05. The analysis results were not adjusted for multiple comparisons. All analyses were conducted using SAS 9.3 (Cary, NC) and R-studio (Boston, MA).

## Results

**Figure 1:**
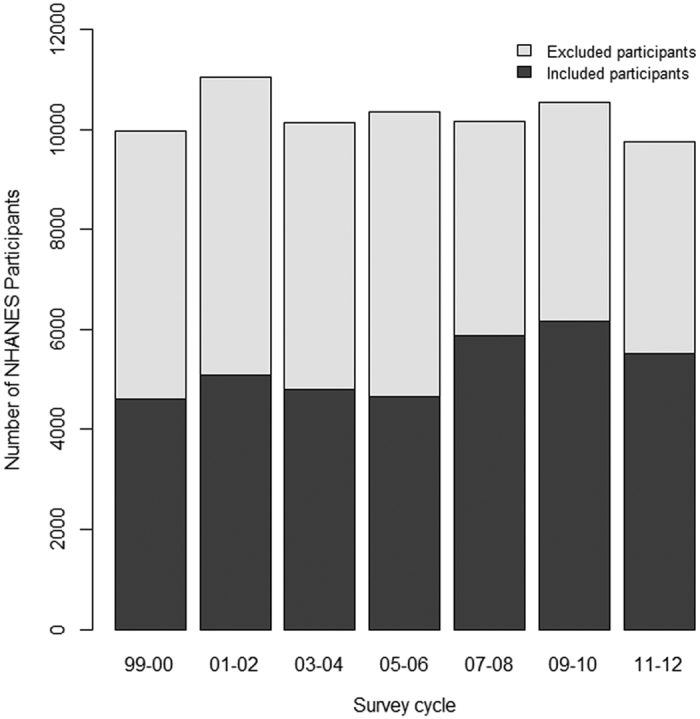
Number of NHANES participants by survey cycle in 1999–2012.

NHANES surveyed a total of 71,916 participants from 1999 to 2012, and 36,673 were included in the analysis. Figure 1 shows the number of NHANES participants and the participants who met the inclusion criteria for the 2-year survey cycle. The average sample size was 5,239 per survey cycle (range 4,597–6,150). The mean age was 50.7 years, 49.8% were male, 47.5% were non-Hispanic white and 20.8% were non-Hispanic black, 46.3% received above high-school diploma education, 59% were married or living with partners, and 30.8% were below the poverty ratio based on the SNAP criteria (Table 1). Overall, there was little change in the demographic and social-economic characteristics of the survey participants over time. Obesity (BMI ≥30 kg/m^2^) increased from 30.4% in 1999–2000 to 38.1% in 2007–2008, and then decreased to 32.3% in 2011–2012.

The overall prevalence of concurrent hypertension, hypercholesterolemia and diabetes increased significantly over the study period, from 3% (95% CI; 2.3%, 3.8%) in 1999–2000 to 6.3% (95% CI; 5.3%, 7.3%) in 2011–2012 (*P* < 0.001 for linear trend while adjusting for demographic characteristics, Table 2). The prevalence almost doubled in both gender groups. It was stable and below 1% among young adults (age <40 years), but increased 107% and 73% in the 40–60 and 60+ year groups, respectively (*P* < 0.01 for trends). After 2009, the prevalence reached above 14% for the 60+ year group. The increasing trend was also found in all race and education categories, except no statistical significance was established for the groups of other Hispanics and non-Hispanic others. The non-Hispanic black group exhibited a doubling in prevalence from 1999 to 2008 and stayed above 10% since then. While the prevalence did not reach a statistically significant increase in the overweight group (*P* = 0.64 for trend) after adjusting for other characteristics, it increased from 0.6% to 2.2% in the normal BMI group (*P* = 0.003 for trend) and from 5.6% to 11.9% in the obesity group (*P* < 0.001 for trend).

Slope analyses of the subgroups revealed, that only BMI showed a significant correlation (*P* < 0.001). The overall prevalence rates of the conditions of concurrent hypertension and diabetes, and concurrent hypercholesterolemia and diabetes also increased significantly during the study period (*P* < 0.001 for both trends). They increased, respectively, from 4.8% (95% CI; 3.7%, 5.9%) and 5.2% (95% CI; 4.1%, 6.2%) in 1999–2000 to 8.1% (95% CI; 6.9%, 9.3%) and 9.0% (95% CI; 7.7%, 10.3%) in 2011–2012 (Supplementary Tables 1 and 2). The significance increments were also detected in most subgroups. The prevalence of concurrent hypertension and diabetes doubled from 4.2% in 1999–2000 to 8.4% in 2011–2012 among male adults while female adults showed a doubling in the prevalence of current hypercholesterolemia and diabetes (4.6% to 9.2%) during the same period. Both combinations were over 15% in 2011–2012 in the obesity group. On the other hand, the overall prevalence of concurrent hypertension and hypercholesterolemia was stable over the study period, which was, however, much higher than the rates of the other two combinations. The prevalence was 19.9% (95% CI; 17.5%, 22.4%) in 1999–2000, 22.6% (95% CI; 22.5%, 24.7%) in 2003–2004 and 23.1% (95% CI; 20.4%, 25.8%) in 2011–2012. The *P*-value was 0.73 for testing a linear trend after adjusting for participant covariates. The prevalence was also stable and remained high in all subgroups (Supplementary Table 3).

The overall medical treatment rate of concurrent hypertension, hypercholesterolemia and diabetes was 69.8% (95% CI; 64.3%, 75.3%) in 1999–2006 and increased to 82.4% (95% CI; 78.9%, 85.8%) in 2007–2012 (*P* = 0.002 for trend, Table 3). Treatment also improved significantly in males <60 years old, non-Hispanic white, other Hispanic, high income, highly educated, unmarried or obese participants. Treatment did not change among non-Hispanic blacks, and decreased among participants with a BMI <25 Kg/m^2^. Overall treatment of the combination of hypertension and diabetes improved from 73.6% in 199–2006 to 79.2% in 2007–2012 (*P* = 0.01 for trend, Supplementary Table 4). Treatment of the combination of hypercholesterolemia and diabetes also improved but did not reach statistical significance (*P* = 0.08). For these combinations of two conditions, treatment generally improved in males, non-Hispanic white and obese participants (Supplementary Table 4). No significant improvement was found for the treatment of the combination of hypertension and hypercholesterolemia (*P* = 0.41), which was, however, already close to 90% during the study period. The treatment also did not show a significant increase in all the subgroups except for participants with a low education level.

Simultaneous goal achievement of diabetes mellitus, hypertension, and hypercholesterolemia was significantly improved over time, from 7.3% (95% CI; 4.7%, 10%) in 1999–2006 to 14.2% (95% CI; 10.8%, 17.5%) in 2007–2012 (*P* = 0.03 for a trend, Table 4). Goal attainments also improved significantly in Mexican Americans, non-Hispanic blacks, low income and low education groups. For combinations of two conditions, simultaneous treatment goal achievements improved for hypercholesterolemia with diabetes or hypertension but not for hypertension and diabetes (Supplementary Table 5). The control rate of hypertension and hypercholesterolemia improved in all subgroups.

## Discussion

From our study, there was a significant increase from 3% to 6.3% in the prevalence of a combination of diabetes, hypertension and hypercholesterolemia between 1999 and 2012 among the U.S. adult population. This finding suggests that more than 12 million adults were living with these three conditions simultaneously in 2012. Among these conditions, a rapid increase in the prevalence of diabetes was becoming a driving force among the major risk factors for cardiovascular disease. From our analysis, significant increment in prevalence could be seen in any concurrent situations with diabetes, while the prevalence of hypertension combined with hypercholesterolemia remained stably high, without significant increment during the last fourteen years, due to a high baseline level.

There are considerable disparities revealed by demographic and social-economic factors. Significantly increased prevalence was found in subgroups including both gender, middle and older age groups (>40 years), non-Hispanic White and non-Hispanic black, and BMI >30 kg/m^2^ in groups with any combination of diabetes. Specifically, in 2011–2012, 14.7% of the elderly group (60+ years) had all three conditions, compared with 6.1% in the 20–60 year old group. The elderly group clearly had poorer treatment achievements, suggesting a heavy and increasing burden on healthcare given the aging population. Better approaches for multiple conditions are urgently needed for the growing elderly population. It was found that SES (social economic status, including education and income level) was inversely correlated with the prevalence of cardiovascular risk factors. The higher the SES status the lower risk was. Nevertheless, the prevalence of risk factors in groups with any combination of diabetes increased in the past fourteen years within all SES subgroups. A better understanding of the reasons for these differences may lead to novel public health prevention programs.

With the rapid development of diabetes, the medicine treatment rate was increased significantly during the past fourteen years. Generally, the treatment rate for all comorbid situations grew from <70% to >80%. Significant increments were found in male patients, age <60 years, non-Hispanic White, and those with a higher SES and BMI. On the other hand, those with higher age (>60 years) and a lower SES had more medical cost coverages than before, though without statistical significance, suggesting more effort might be needed for those subgroups.

In sub-analysis for different combinations of the three major risk factors, a similar trend was found. The most significant increase was found in a combination of hypertension and diabetes, hypercholesterolemia and diabetes especially in males non-Hispanic White BMI >30 kg/m^2^, while we did not find a significant increment in medical treatment rates for hypertension and hypercholesterolemia, due to the higher cost coverage level at baseline. The general medical treatment rates in subgroups, with any combination of factors with diabetes, were around 79% in 2007–2012, although significantly higher than in 1999–2006, but still lower compared to 90.7% for the medicine treatment rate of hypertension and hypercholesterolemia in 2007–2012. These findings imply that more treatment cost coverage for diabetes should be implemented in order to better improve the current status of the rapidly growing diabetic population.

Although 82.4% of subjects with three conditions combined had medicine treatment cost coverage, only 14.2% of them had reached the target goals, meaning that the majority did not succeed in reducing their cardiovascular risks well enough. In a sub-analysis for those with any of the two conditions, the goal achievement rates were around 17–24.5%. Generally speaking, the goal attainment rates for all these cardiovascular risks were far from satisfactory. We detected a statistically significant increment in successful medication control rates in two subgroups with hypercholesterolemia. However, only 25% of subjects with hypertension and hypercholesterolemia reached their treatment goal, 20% in the hypertension and diabetes group and <20% in the hypercholesterolemia and diabetes group.

In conclusion, due to the rapid development of diabetes, the cardiovascular risks associated with this condition have increased accordingly in any combination of disease with diabetes, while no dramatic growth was found in subjects who did not have diabetes. As a result, the treatment cost coverage has lagged far behind especially in the groups with diabetes. More strikingly, treatment goal attainment rates in any groups with combination of diabetes were even lower than in those groups without diabetes.

The main strength of this study was the use of a large, continuous, national representative survey. All survey measurements and data were collected with standardized methods over time. The findings in this paper are subject to several limitations. First, we did not distinguish between undiagnosed and diagnosed conditions. Second, definitions of these conditions are continuously changing and may also be stratified. For example, hypertension could be defined as BP ≥130/80 mmHg for diabetic subjects^36^. The new guidelines from the Eight Joint National Committee on High Blood Pressure recommends treating hypertension for 60+ year old adults with a BP ≥150/90 mmHg^43^. Finally, the study data rely on self-reported information and may be subject to recall and social desirability bias.

In summary, more intensive treatment regimens are needed for patients with a combination of diabetes, hypertension and hypercholesterolemia, in order to curb the cardiovascular endpoints expected in the near future, as the majority of them did not meet their treatment targets. As an urgent priority, treatment adjustment or intensification for diabetic patients may be required as the treatment goal achievement rate of these subjects was even lower compared to those without diabetes.

## Additional Information

**How to cite this article**: Song, Y. *et al.* Increasing trend of diabetes combined with hypertension or hypercholesterolemia: NHANES data analysis 1999–2012. *Sci. Rep.* **6**, 36093; doi: 10.1038/srep36093 (2016).

**Publisher’s note**: Springer Nature remains neutral with regard to jurisdictional claims in published maps and institutional affiliations.

## Supplementary Material

Supplementary Information

## Figures and Tables

**Table 1:** NHANES participants characteristics from 1999 to 2012.

		All (N = 36,673)	1999–2010 (N = 5,094)	2001–2002 (N = 4,808)	2003–2004 (N = 4,673)	2005–2006 (N = 5,878)	2007–2008 (N = 6,150)	2009–2010 (N = 5,503)	2011–2012 (N = 4,597)
Gender N (%)	Male	18,266 (49.8%)	2,536 (49.8%)	2,418 (50.3%)	2,387 (51.4%)	2,910 (49.5%)	3,006 (48.9%)	2,740 (49.8%)	2,269 (49.4%)
	Female	18,407 (50.2%)	2,558 (50.2%)	2,390 (49.7%)	2,256 (48.6%)	2,968 (50.5%)	3,144 (51.1%)	2,763 (50.2%)	2,328 (50.6%)
Age (years)	Mean (SD)	50.7 (18.6)	51.4 (19.2)	51.9 (19.5)	49.9 (18.9)	51.0 (17.9)	49.9 (17.9)	49.1 (17.9)	51.9 (18.9)
Race/Ethnicity N (%)	Mexican American	6,723 (18.3%)	1,038 (20.4%)	923 (19.2%)	897 (19.3%)	1,011 (17.2%)	1,120 (18.2%)	534 (9.7%)	1,200 (26.1%)
	Other Hispanic	2,651 (7.2%)	220 (4.3%)	147 (3.1%)	140 (3%)	657 (11.2%)	628 (10.2%)	573 (10.4%)	286 (6.2%)
	Non-Hispanic White	1,7430 (47.5%)	2,699 (53%)	2,573 (53.5%)	2,345 (50.5%)	2,752 (46.8%)	2,953 (48%)	2,023 (36.8%)	2,085 (45.4%)
	Non-Hispanic Black	7,644 (20.8%)	972 (19.1%)	956 (19.9%)	1,077 (23.2%)	1,214 (20.7%)	1109 (18%)	1,437 (26.1%)	879 (19.1%)
	Other	2225 (6.1%)	165 (3.2%)	209 (4.3%)	184 (4%)	244 (4.2%)	340 (5.5%)	936 (17%)	147 (3.2%)
Education N (%)	<high school	11,065 (30.2%)	1,589 (31.3%)	1,430 (29.8%)	1,295 (27.9%)	1,845 (31.4%)	1,755 (28.6%)	1,325 (24.1%)	1,826 (39.9%)
	High school	8,577 (23.4%)	1197 (23.6%)	1,222 (25.5%)	1112 (24%)	1,444 (24.6%)	1,413 (23%)	1,161 (21.1%)	1,028 (22.5%)
	>high school	16,938 (46.3%)	2,287 (45.1%)	2,143 (44.7%)	2,227 (48.1%)	2,582 (44%)	2,967 (48.4%)	3,012 (54.8%)	1,720 (37.6%)
Marital status N (%)	Married/Living with partners	21,339 (59%)	3,092 (60.8%)	2,806 (58.4%)	2,812 (60.7%)	3,476 (59.2%)	3,622 (58.9%)	3,082 (56.1%)	2,449 (59.8%)
	Other	14,801 (41%)	1,994 (39.2%)	1,999 (41.6%)	1,824 (39.3%)	2,398 (40.8%)	2,525 (41.1%)	2,414 (43.9%)	1,647 (40.2%)
Income to poverty ratio^1^	≤1.3	10,266 (30.8%)	1,279 (27.4%)	1,279 (28.4%)	1,135 (25.8%)	1,641 (30.8%)	1,872 (33.8%)	1,843 (36.8%)	1,217 (31.3%)
	1.3–3.5	12,689 (38.1%)	1,796 (38.4%)	1,831 (40.7%)	1,761 (40%)	2,076 (39%)	2,080 (37.6%)	1,670 (33.3%)	1,475 (37.9%)
	>3.5	10,385 (31.1%)	1,598 (34.2%)	1,391 (30.9%)	1,511 (34.3%)	1,605 (30.2%)	1,585 (28.6%)	1,498 (29.9%)	1,197 (30.8%)
Systolic BP (mmHg)	Mean (SD)	125.2 (20.0)	126.4 (21.1)	126.4 (21.4)	125.2 (19.8)	124.8 (19.0)	123.0 (18.6)	123.7 (18.5)	128.2 (21.5)
Diastolic BP (mmHg)	Mean (SD)	70.4 (13.5)	71.5 (13.8)	69.8 (13.8)	69.7 (13.6)	70.1 (13.1)	69.3 (13.1)	71.0 (12.6)	71.8 (14.4)
BMI (Kg/m^2^)	Mean (SD)	28.7 (6.6)	28.2 (6.2)	28.4 (6.3)	28.8 (6.7)	29.0 (6.7)	29.1 (6.8)	28.8 (6.9)	28.4 (6.2)
Obesity N (%)	BMI ≥30	11,789 (34.7%)	1,340 (30.4%)	1,451 (32.7%)	1,532 (35.2%)	2,028 (36.5%)	2,255 (38.1%)	1,855 (35.8%)	1,328 (32.3%)
HbA1c	Mean (SD)	5.7 (1.1)	5.6 (1.1)	5.6 (1.0)	5.6 (1.0)	5.8 (1.1)	5.8 (1.0)	5.8 (1.1)	5.6 (1.2)
Total cholesterol mg/dL	Mean (SD)	198.2 (42.2)	202.5 (43.1)	201.3 (43.8)	196.7 (42.0)	196.9 (42.0)	195.4 (41.3)	192.9 (41.5)	203.9 (40.7)

Data are presented as Number (%) for categorical variables and mean (SD) for continuous variables. ^1^Income-to-poverty ratio was categorized according to the Supplemental Nutrition Assistance Program (SNAP) criteria.

**Table 2:** Prevalence (%) of adults with concurrent hypercholesterolemia, diabetes and hypertension in the United States from 1999 to 2012.

		Prevalence as percentage (95% confidence interval)							*P* for trend^2^	P for slopes between subgroups
		1999–2010 (N = 3, 847)^1^	2001–2002 (N = 4, 303)	2003–2004 (N = 4, 143)	2005–2006 (N = 4, 082)	2007–2008 (N = 5, 204)	2009–2010 (N = 5, 547)	2011–2012 (N = 4, 815)		
Overall		3.0 (2.3, 3.8)	3.5 (2.9, 4.1)	4.8 (4, 5.7)	4.6 (3.8, 5.4)	5.5 (4.6, 6.4)	6.0 (5.3, 6.6)	6.3 (5.3, 7.3)	<0.001	
Gender	Male	2.8 (1.6, 4.1)	3.2 (2.3, 4)	4.4 (3.3, 5.5)	3.9 (3.1, 4.7)	5.0 (4, 6)	5.9 (4.9, 7)	6.4 (5.2, 7.7)	<0.001	0.145
	Female	3.2 (2.5, 4)	3.9 (3.2, 4.6)	5.3 (4.4, 6.2)	5.3 (4.1, 6.5)	6.0 (4.4, 7.5)	6.0 (5, 7)	6.2 (4.9, 7.4)	<0.001	
Age (years)	[20, 40]	0.3 (–0.3, 1)	0.3 (–0.2, 0.9)	0.4 (0.1, 0.7)	0.6 (0.2, 0.9)	0.7 (0.3, 1.2)	0.5 (0.2, 0.8)	0.3 (0, 0.7)	0.490	0.693
	[40, 60]	2.8 (1.6, 4)	3.2 (2, 4.3)	4.6 (3.7, 5.5)	4.1 (2.9, 5.4)	5.9 (4.8, 7.1)	5.4 (4.2, 6.6)	5.8 (3.9, 7.7)	0.003	
	60+	8.5 (7.2, 9.9)	10 (8.8, 11.3)	12.4 (10, 14.8)	11.5 (10, 13)	11.8 (9.2, 14.4)	14.3 (12.8, 15.7)	14.7 (12.7, 16.7)	<0.001	
Race / Ethnicity	Mexican American	3.3 (2.2, 4.3)	3.1 (2.2, 3.9)	3.7 (2.3, 5.1)	4.7 (2.4, 7.1)	4.8 (3.3, 6.3)	6.7 (4.4, 8.9)	6.1 (2.5, 9.7)	0.020	0.837
	Other Hispanic	2.9 (0.6, 5.1)	4.0 (1, 6.9)	5.1 (–2.8, 13.1)	5.4 (0.9, 9.9)	5.1 (3.7, 6.4)	6.0 (3, 8.9)	6.6 (4.9, 8.3)	0.220	
	Non-Hispanic White	2.7 (1.7, 3.7)	3.1 (2.6, 3.6)	4.5 (3.6, 5.4)	4.0 (3.2, 4.9)	4.8 (3.6, 6.1)	5.3 (4.4, 6.1)	5.6 (4.4, 6.8)	<0.001	
	Non-Hispanic Black	5.5 (3.8, 7.2)	6.6 (4.4, 8.8)	6.4 (5, 7.8)	7.9 (5.4, 10.5)	11.2 (8.8, 13.7)	10.6 (8.5, 12.8)	10.2 (8.3, 12.1)	<0.000	
	Other	2.6 (0, 5.1)	4.2 (–0.5, 8.9)	8.5 (3, 13.9)	5.2 (1.5, 8.8)	5 (1.7, 8.4)	5.2 (2.5, 7.9)	7.3 (3.3, 11.3)	0.450	
Education level	<High school	6.7 (5, 8.4)	6.6 (4.3, 8.9)	7.8 (5.2, 10.4)	6.6 (4.9, 8.3)	8.7 (7.2, 10.2)	10.1 (8.6, 11.5)	9.5 (6.1, 12.8)	0.060	0.195
	High school	2.4 (1.4, 3.5)	4.1 (2.9, 5.4)	5.3 (3.8, 6.8)	6.1 (4.4, 7.9)	6.5 (4.2, 8.9)	5.5 (4, 7.1)	7.4 (4.9, 10)	0.010	
	>High school	1.6 (0.5, 2.6)	2.2 (1.5, 3)	3.7 (2.9, 4.4)	3.4 (2.4, 4.3)	3.9 (3.1,. 4.7)	4.8 (3.7, 6)	5.1 (3.7, 6.6)	<0.001	
Income to poverty ratio	≤1.3	4.0 (2.7, 5.4)	5.2 (3.7, 6.7)	6.2 (4.6, 7.9)	7.6 (4.7, 10.4)	7.2 (6, 8.5)	6.6 (4.8, 8.3)	7.6 (5, 10.2)	0.006	0.311
	1.3–3.5	3.6 (2.1, 5.1)	3.8 (2.9, 4.7)	4.9 (3.5, 6.3)	5.1 (3.7, 6.5)	6.7 (5.7, 7.6)	7.5 (6.1, 9)	7.6 (5.9, 9.3)	<0.001	
	>3.5	1.7 (0.6, 2.8)	2.5 (1.7, 3.4)	3.8 (2.5, 5.1)	2.9 (1.9, 3.8)	4.0 (3, 5)	4.3 (2.9, 5.6)	4.5 (2.3, 6.7)	0.060	
Marital status	Married/living with partner	3.1 (1.9, 4.2)	3.1 (2.2, 4)	4.4 (3.5, 5.3)	4.2 (3.3, 5.2)	5.3 (4.1, 6.4)	5.5 (4.6, 6.5)	5.9 (4.8, 6.9)	<0.001	0.808
	Other	2.5 (1.7, 3.3)	4.3 (3, 5.5)	5.7 (4.4, 7.1)	5.4 (4.2, 6.5)	6.0 (4.8, 7.1)	6.5 (5.6, 7.5)	7.0 (5.3, 8.6)	<0.001	
BMI (kg/m^2^)	<25	0.6 (0.1, 1.1)	1.1 (0.6, 1.5)	1.5 (1, 2)	1.5 (0.8, 2.1)	2.2 (1.3, 3.1)	1.2 (0.7, 1.7)	2.2 (1.2, 3.2)	0.003	<0.001
	25–29	3.0 (2.1, 3.8)	3.4 (2.6, 4.3)	4.1 (2.4, 5.8)	3.9 (2.4, 5.4)	3.2 (2.3, 4.1)	3.7 (2.7, 4.8)	3.8 (3, 4.5)	0.640	
	30+	5.6 (4.1, 7)	5.9 (4.4, 7.3)	9.1 (7.1, 11.2)	8.1 (6.6, 9.6)	10.9 (8.9, 12.9)	11.8 (10.1, 13.5)	11.9 (9.5, 14.3)	<0.001	

Note: Data are presented as percentages (95% confidence interval), unless otherwise indicated. ^1^N is the overall sample size; ^2^The overall *P*-value was adjusted for all characteristics and the *P*-value for each subgroup was adjusted for the rest of the characteristics.

**Table 3:** Percent of simultaneous treatment for hypertension, hypercholesterolemia and diabetes among adult participants with these three conditions between 1999 and 2012.

		Percentage (95% CI)		*P*^1^
		1999–2006N = 572	2007–2012N = 1, 025	
Overall		69.8 (64.3, 75.3)	82.4 (78.9, 85.8)	0.002
Gender	Male	65.9 (56.7, 75.1)	82.1 (77.8, 86.5)	0.001
	Female	72.9 (65.7, 80.2)	82.6 (77.8,. 87.4)	0.120
Age (years)	[20, 40]	82.5 (57.2, 107.7)	93.9 (85.8, 102)	<0.001
	[40, 60]	59.3 (48.8, 69.9)	80.8 (74.7, 86.9)	0.003
	60+	75.6 (69.8, 81.4)	82.8 (79.3, 86.3)	0.150
Race	Mexican American	82.0 (74.7, 89.3)	88.6 (81.9, 95.2)	0.370
	Other Hispanic	51.1 (24.5, 77.7)	76.2 (68.9, 83.5)	0.004
	Non-Hispanic White	67.8 (60.9, 74.7)	84.2 (79.5, 88.8)	0.001
	Non-Hispanic Black	79.3 (70.4, 88.2)	79.7 (74.8, 84.6)	0.670
	Other	74.3 (58.4, 90.2)	72.1 (55.0, 89.2)	0.350
Education	<High school	79.2 (72.7, 85.7)	81.6 (76.5, 86.6)	0.890
	High school	64.9 (54.5, 75.3)	79.7 (74.3, 85.2)	0.060
	>High school	66.0 (56.9, 75.1)	84.2 (78.5, 89.9)	0.004
Income to poverty ratio	≤1.3	76.7 (66.6, 86.8)	84.5 (80.0, 89.0)	0.190
	1.3–3.5	71.5 (62.4, 80.7)	78.1 (72.5, 83.6)	0.390
	>3.5	61.3 (49.4, 73.2)	84.7 (78.4, 91.0)	0.005
Marital status	Married/living with partner	70.5 (63.2, 77.8)	81.5 (76.8, 86.1)	0.060
	Other	69.4 (60.3, 78.5)	83.6 (79.4, 87.8)	0.020
BMI (kg/m^2^)	<25	80.3 (68.2, 92.4)	75.7 (61.8, 89.6)	0.200
	25–29	67.5 (56.2, 78.9)	76.6 (70.1, 83.2)	0.470
	30+	70.5 (65.0, 76.0)	84.3 (80.9, 87.7)	<0.001

Note: Data are presented as percentages (95% confidence interval), unless otherwise indicated. ^1^For comparing 2007–2012 with 1999–2006, the data were adjusted for participant characteristics.

**Table 4:** Percent of simultaneous control of hypertension, hypercholesterolemia and diabetes among adult participants with these three conditions between 1999 and 2012.

		Percentage (95% CI)		
		1999–2006N = 928	2007–2012N = 1, 228	*P*^1^
Overall		7.3 (4.7, 10)	14.2 (10.8, 17.5)	0.030
Gender	Male	10.9 (5.6, 16.2)	15.2 (10.8, 19.6)	0.640
	Female	4.5 (1.8, 7.2)	13.2 (8.8, 17.7)	0.006
Age (yrs)	[20, 40]	0.0 (0, 0)	26 (2.9, 49)	<0.001
	[40, 60]	6.7 (2, 11.5)	16.5 (10.5, 22.5)	0.030
	60+	8.1 (5.3, 10.9)	12.1 (8.8, 15.5)	0.470
Race	Mexican American	1.0 (−0.2, 2.2)	7.3 (4, 10.7)	0.006
	Other Hispanic	6.4 (−4.2, 16.9)	11.8 (6.9, 16.6)	0.670
	Non-Hispanic White	8.5 (4.9, 12.1)	16.9 (12, 21.7)	0.060
	Non-Hispanic Black	4.8 (2, 7.7)	11.4 (8.5, 14.4)	0.020
	Other	9.7 (−0.9, 20.3)	8.4 (−0.4, 17.2)	0.500
Education	<High school	3.8 (1.6, 6)	10.2 (6.7, 13.7)	0.030
	High school	7.7 (3.3, 12)	12.8 (7.3, 18.3)	0.210
	>high school	10.1 (4.9, 15.4)	17.5 (12.2, 22.8)	0.210
Income to poverty ratio	≤1.3	4.3 (1.2, 7.3)	10 (6.5, 13.5)	0.030
	1.3–3.5	6 (2.7, 9.3)	14.5 (9, 20)	0.030
	>3.5	13.7 (6.6, 20.8)	17.4 (10.5, 24.4)	0.530
Marital status	Married/living with partner	9.0 (4.9, 13.1)	16.4 (12.2, 20.5)	0.110
	Other	5.5 (2.7, 8.2)	11.0 (6.8, 15.1)	0.080
BMI (kg/m^2^)	<25	10.5 (1.8, 19.2)	12.0 (3.2, 20.9)	0.340
	25–29	4.5 (1.7, 7.3)	13.0 (7.3, 18.7)	0.010
	30+	8.6 (4.6, 12.6)	14.6 (10.9, 18.4)	0.100

Note: Data are presented as percentages (95% confidence interval), unless otherwise indicated. ^1^For comparing 2007–2012 with 1999–2006, the data were adjusted for participant characteristics.
