# Supplementary material for: Increasing trend of diabetes combined with hypertension or hypercholesterolemia: NHANES data analysis 1999–2012
Source: Sci Rep. 2016 Nov 2;6:36093. doi: 10.1038/srep36093 (PMC5090961; doi:10.1038/srep36093)
Supplement: Supplementary Information [file srep36093-s1.doc]

**Increasing trend of** **diabetes combined with hypertension or hypercholesterolemia: NHANES data analysis 1999-2012**

Yongfeng Song^1*^, Xiaoyun Liu^2*^, Xiaolin Zhu^3^, Bin Zhao^3^, Bo Hu^4^, Xia Sheng^3^, Lan Chen^3^, Miao Yu^5^, Tao Yang^2^, Jiajun Zhao^1#^

**Supplementary Table 1.** Prevalence (%) of US adults with concurrent hypertension and diabetes between 1999 and 2012

|  |  | Prevalence as percentage (95% confidence interval) | | | | | | | *P* for trend^1^ |
| --- | --- | --- | --- | --- | --- | --- | --- | --- | --- |
|  |  | 1999–2010  (N = 4,081) | 2001–2002  N = 4,548 | 2003–2004  N = 4,309 | 2005–2006  N = 4,283 | 2007–2008  N = 5,460 | 2009–2010  N = 5,798 | 2011–2012  N = 5,083 |  |
| Overall |  | 4.8 (3.7,5.9) | 5.2 (4.4,5.9) | 6.6 (5.5,7.6) | 6.5 (5.7,7.4) | 7.6 (6.2,8.9) | 8.1 (7.4,8.7) | 8.1 (6.9,9.3) | < 0.001 |
| Gender | Male | 4.2 (3,5.4) | 4.9 (3.7,6.1) | 6.0 (4.9,7.1) | 5.9 (5.1,6.7) | 7.0 (5.7,8.4) | 7.9 (6.8,9) | 8.4 (6.6,10.2) | < 0.001 |
|  | Female | 5.3 (4,6.6) | 5.4 (4.6,6.1) | 7.1 (5.8,8.4) | 7.1 (5.7,8.5) | 8.1 (6.3,9.8) | 8.2 (7.2,9.2) | 7.7 (6.6,8.9) | 0.002 |
| Age (yrs) | [20, 40] | 0.8 (0.1,1.5) | 0.7 (0.1,1.3) | 0.7 (0.2,1.2) | 1.1 (0.4,1.8) | 0.8 (0.4,1.2) | 0.9 (0.4,1.3) | 0.9 (0.3,1.5) | 0.490 |
|  | [40, 60] | 4.3 (2.6,6.1) | 4.7 (3.3,6.1) | 6.6 (5,8.1) | 5.5 (4.2,6.7) | 7.5 (6.1,8.9) | 6.8 (5.8,7.8) | 7.7 (5.3,10) | 0.040 |
|  | 60+ | 13.1 (11.2,15) | 14.2 (13,15.5) | 16.1 (13,19.2) | 16.6 (14,19.3) | 17.8 (13.7,21.8) | 20 (18.2,21.8) | 18.0 (15.9,20.2) | < 0.001 |
| Race / Ethnicity | Mexican American | 4.8 (3.2,6.3) | 4.2 (3.1,5.3) | 5.9 (4.2,7.6) | 6.3 (3.8,8.7) | 6.1 (4,8.2) | 8.4 (5.8,11.1) | 7.7 (4.3,11.2) | 0.070 |
|  | Other Hispanic | 6 (1.9,10.1) | 5.7 (2,9.3) | 6.7 (-3.3,16.8) | 5.5 (0.8,10.1) | 7.1 (5.2,9) | 7.3 (4.3,10.3) | 7.5 (5.3,9.6) | 0.860 |
|  | Non-Hispanic White | 3.9 (2.7,5.2) | 4.6 (4,5.1) | 5.9 (4.8,7) | 5.7 (4.8,6.6) | 6.9 (5,8.8) | 7.0 (6.1,8) | 7.2 (5.8,8.5) | < 0.001 |
|  | Non-Hispanic Black | 8.8 (6.8,10.8) | 9.9 (7.1,12.6) | 9.4 (7.4,11.5) | 12.4 (9.5,15.3) | 13.8 (11,16.6) | 13.6 (11.1,16.1) | 13.5 (11,16) | 0.006 |
|  | Other | 6.4 (0.7,12) | 4.7 (-0.2,9.6) | 10.6 (4.9,16.3) | 6.5 (2.8,10.1) | 6.3 (2.8,9.9) | 9.4 (6.3,12.5) | 8.4 (4.2,12.6) | 0.660 |
| Education level | < high school | 9.6 (7.5,11.8) | 9.6 (7.2,12.1) | 10.6 (7.8,13.5) | 10.3 (8.1,12.5) | 12.1 (10.5,13.8) | 13.7 (11.9,15.5) | 12.5 (8.8,16.2) | 0.055 |
|  | High school | 4.7 (3.4,6) | 5.5 (4.2,6.8) | 6.6 (5,8.1) | 8.5 (6.8,10.1) | 8.8 (6,11.6) | 7.8 (6.4,9.3) | 9.8 (7.2,12.5) | 0.020 |
|  | > high school | 2.5 (1.2,3.8) | 3.5 (2.5,4.5) | 5.2 (4,6.5) | 4.5 (3.4,5.7) | 5.3 (4.1,6.5) | 6.3 (5.1,7.5) | 6.3 (4.9,7.8) | 0.002 |
| Income to poverty ratio | <=1.3 | 7.1 (4.5,9.7) | 7.5 (5.8,9.2) | 8.3 (6.5,10.1) | 9.6 (7.2,12) | 10 (7.7,12.2) | 9.3 (7,11.6) | 9.4 (6.5,12.2) | 0.060 |
|  | 1.3-3.5 | 5.6 (3.7,7.4) | 5.9 (4.6,7.3) | 6.8 (4.9,8.7) | 7.6 (5.8,9.5) | 9.2 (7.5,10.8) | 9.8 (8.3,11.4) | 9.3 (7.6,10.9) | < 0.001 |
|  | >3.5 | 2.4 (1.1,3.7) | 3.4 (2.3,4.5) | 4.9 (3.6,6.2) | 4.0 (2.9,5.2) | 5.2 (3.7,6.6) | 5.7 (4.2,7.3) | 6.2 (3.6,8.9) | 0.020 |
| Marital status | Married/living with partner | 4.5 (3,5.9) | 4.6 (3.3,5.8) | 6.2 (4.9,7.5) | 6.1 (5.1,7.2) | 7.2 (5.6,8.8) | 7.7 (6.9,8.6) | 7.7 (6.6,8.8) | < 0.001 |
|  | Other | 5.1 (3.7,6.6) | 6.2 (4.9,7.6) | 7.2 (5.7,8.7) | 7.3 (6.2,8.4) | 8.3 (6.6,10) | 8.5 (7.5,9.5) | 8.7 (6.7,10.7) | 0.010 |
| BMI (kg/m^2^) | <25 | 1.2 (0.2,2.2) | 1.4 (0.9,2) | 2.1 (1.2,3.1) | 2.3 (1.5,3.1) | 2.9 (1.8,4) | 1.7 (1.2,2.3) | 2.7 (1.6,3.8) | 0.020 |
|  | 25-29 | 4.4 (3,5.7) | 4.3 (3.4,5.3) | 5.2 (3.4,7) | 5.5 (4,6.9) | 4.7 (3.6,5.8) | 5.2 (3.8,6.5) | 5.1 (4.1,6.1) | 0.920 |
|  | 30+ | 9 (6.6,11.3) | 9.1 (7.3,11) | 12.4 (10.4,14.5) | 11.2 (9.3,13.1) | 14.5 (11.7,17.3) | 15.8 (14.1,17.5) | 15.2 (12.9,17.6) | < 0.001 |

Note: Data are presented as percentages (95% confidence interval), unless otherwise indicated.^1^ The overall *P*-value was adjusted for all characteristics and the *P*-value for each subgroup was adjusted according to other characteristics.

**Supplementary Table 2**. Prevalence (%) of US adults with concurrent hypercholesterolemia and diabetes between 1999 and 2012

|  |  | Prevalence as percentage (95% confidence interval) | | | | | | | *P* for trend^1^ |
| --- | --- | --- | --- | --- | --- | --- | --- | --- | --- |
|  |  | 1999–2010  (N = 3,920) | 2001–2002  (N = 4,443) | 2003–2004  (N = 4,323) | 2005–2006  (N = 4,211) | 2007–2008  (N = 5,349) | 2009–2010  (N = 5,710) | 2011–2012  (N = 4,965) |  |
| Overall |  | 5.2 (4.1,6.2) | 5.5 (4.7,6.3) | 7.2 (6.1,8.3) | 6.5 (5.5,7.5) | 7.9 (6.9,9) | 7.7 (7,8.5) | 9.0 (7.7,10.3) | < 0.001 |
| Gender | Male | 5.7 (4,7.4) | 5.6 (4.8,6.5) | 7.3 (5.8,8.8) | 5.8 (4.7,7) | 7.8 (6.7,8.9) | 8.1 (6.9,9.3) | 8.8 (7,10.6) | 0.010 |
|  | Female | 4.6 (3.8,5.5) | 5.4 (4.5,6.4) | 7.1 (6,8.2) | 7.1 (5.6,8.6) | 8.0 (6.2,9.8) | 7.4 (6.3,8.4) | 9.2 (7.4,10.9) | < 0.001 |
| Age (years) | [20, 40] | 0.8 (0.4,1.3) | 1.3 (0.5,2.1) | 1.5 (0.6,2.4) | 1.5 (1,2.1) | 1.8 (1.2,2.4) | 1.4 (1.1,1.8) | 1.2 (0.6,1.9) | 0.340 |
|  | [40, 60] | 5.7 (3.8,7.6) | 5.9 (4.5,7.3) | 8.1 (6.5,9.6) | 6.6 (5,8.2) | 9.1 (7.6,10.5) | 7.5 (6,9) | 9.7 (7.7,11.8) | 0.010 |
|  | 60+ | 12.6 (10.1,15.1) | 12.6 (11,14.2) | 14.9 (12.5,17.3) | 13.7 (11.8,15.5) | 15.1 (11.9,18.3) | 17.0 (15.4,18.5) | 18.1 (15.4,20.9) | < 0.001 |
| Race / Ethnicity | Mexican American | 5.2 (3.6,6.9) | 5.3 (4.7,5.9) | 7.3 (4.9,9.6) | 8.2 (6.6,9.9) | 7.8 (6.3,9.3) | 10.5 (7.8,13.3) | 10.7 (7,14.4) | 0.001 |
|  | Other Hispanic | 4.5 (1.4,7.7) | 8.8 (4.6,13) | 9.6 (0.5,18.7) | 9.6 (3.1,16.1) | 7.5 (5.6,9.4) | 8.4 (6.3,10.5) | 11.1 (9.1,13.1) | 0.210 |
|  | Non-Hispanic White | 4.8 (3.4,6.3) | 4.7 (4,5.4) | 6.6 (5.4,7.7) | 5.5 (4.2,6.7) | 7.0 (5.3,8.6) | 6.8 (5.9,7.8) | 7.5 (5.9,9.1) | 0.001 |
|  | Non-Hispanic Black | 7.2 (5.4,9) | 7.3 (5.7,9) | 8.5 (6.8,10.2) | 10.1 (7.8,12.4) | 13.5 (11.5,15.4) | 11.4 (9.5,13.4) | 13.8 (11,16.6) | < 0.001 |
|  | Other | 6.8 (1.6,11.9) | 11.5 (4.5,18.4) | 11.6 (4.8,18.3) | 8.1 (4.6,11.5) | 10.0 (5.2,14.7) | 7.2 (3.8,10.7) | 11.6 (6.2,17) | 0.980 |
| Education level | < High school | 9.8 (7.2,12.3) | 8.8 (6.8,10.7) | 12 (8.8,15.2) | 9.2 (7.6,10.8) | 11.4 (9.7,13.1) | 12.5 (10.8,14.2) | 14.4 (11.2,17.6) | 0.030 |
|  | High school | 5.1 (3.3,7) | 5.8 (4.4,7.2) | 7.1 (5.7,8.5) | 7.8 (5.5,10) | 8.9 (6.2,11.6) | 7.2 (5.4,8.9) | 11.3 (8.2,14.4) | 0.010 |
|  | > High school | 3 (1.7,4.3) | 4.3 (3.2,5.4) | 5.7 (4.5,6.9) | 5.1 (3.8,6.4) | 6.2 (5,7.4) | 6.4 (5.4,7.5) | 6.8 (5.3,8.4) | < 0.001 |
| Income to poverty ratio | ≤ 1.3 | 6.3 (4.3,8.3) | 7.0 (5.5,8.6) | 8.7 (7,10.4) | 9.1 (6.1,12) | 9.5 (7.7,11.3) | 8.6 (7,10.3) | 12.1 (9.2,14.9) | < 0.001 |
|  | 1.3–3.5 | 6.5 (4.9,8.2) | 5.8 (4.5,7) | 7.3 (5.2,9.5) | 7.6 (6.1,9.2) | 8.7 (7.5,9.9) | 9.3 (7.5,11.1) | 10.4 (8.6,12.1) | < 0.001 |
|  | > 3.5 | 2.8 (1.3,4.3) | 4.2 (2.9,5.5) | 6.0 (4.7,7.2) | 4.1 (2.8,5.5) | 6.7 (5,8.3) | 5.9 (4.5,7.3) | 6.1 (3.7,8.4) | 0.150 |
| Marital status | Married/living with partner | 5.5 (3.8,7.2) | 5.1 (3.9,6.3) | 6.9 (5.5,8.4) | 6.2 (5.2,7.3) | 8.0 (6.6,9.5) | 7.3 (6.2,8.3) | 8.4 (7.2,9.6) | 0.003 |
|  | Other | 4.1 (2.6,5.6) | 6.3 (4.6,8.1) | 7.7 (6.3,9.1) | 7.0 (5.5,8.4) | 7.7 (6.8,8.7) | 8.4 (7.3,9.4) | 9.9 (7.9,11.8) | < 0.001 |
| BMI (kg/m^2^) | < 25 | 2.1 (1.1,3.1) | 2.4 (1.3,3.5) | 3.0 (1.4,4.6) | 2.5 (1.7,3.3) | 3.1 (2.1,4.2) | 2.2 (1.4,3.1) | 4.1 (2.5,5.7) | 0.080 |
|  | 25–29 | 5.0 (3.4,6.6) | 5.5 (4.4,6.5) | 5.8 (3.6,7.9) | 5.5 (3.9,7.2) | 4.9 (3.9,6) | 5.5 (4.1,6.9) | 5.7 (4.7,6.6) | 0.360 |
|  | 30+ | 8.6 (6.7,10.6) | 8.8 (6.5,11.1) | 13.2 (10.8,15.5) | 11 (9.1,12.9) | 15.2 (12.6,17.9) | 14.3 (12.6,15.9) | 16.1 (13.1,19.1) | < 0.001 |

Note: Data are presented as percentages (95% confidence interval), unless otherwise indicated.^1^The overall *P*-value was adjusted for all characteristics and the *P*-value for each subgroup was adjusted for the remaining characteristics

**Supplementary Table 3.** Prevalence (%) of US adults with concurrent hypertension and hypercholesterolemia between 1999 and 2012

|  |  | Prevalence as percentage (95% confidence interval) | | | | | | | *P* for trend^1^ |
| --- | --- | --- | --- | --- | --- | --- | --- | --- | --- |
|  |  | 1999–2010  (N = 3,847) | 2001–2002  (N = 4,303) | 2003–2004  (N = 4,143) | 2005–2006  (N = 4,082) | 2007–2008  (N = 5,204) | 2009–2010  (N = 5,547) | 2011–2012  (N = 4,815) |  |
| Overall |  | 19.9 (17.5,22.4) | 19.5 (17.2,21.7) | 22.6 (20.5,24.7) | 22.6 (20.3,24.8) | 22.1 (20.4,23.8) | 22.6 (20.4,24.7) | 23.1 (20.4,25.8) | 0.730 |
| Gender | Male | 18.6 (15.8,21.3) | 16.4 (13.6,19.1) | 21.6 (19,24.3) | 20.6 (18.3,22.9) | 19.9 (18.1,21.8) | 21.5 (18.7,24.2) | 21.9 (18.5,25.4) | 0.540 |
|  | Female | 21.3 (18.2,24.4) | 22.5 (19.9,25.1) | 23.5 (21.1,26) | 24.5 (21.6,27.3) | 24.1 (21.5,26.6) | 23.6 (21.5,25.7) | 24.3 (21.8,26.7) | 0.160 |
| Age (years) | [20, 40] | 3.8 (1.9,5.6) | 4.1 (2.5,5.6) | 4.0 (3.1,5) | 4.2 (2.6,5.8) | 4.4 (2.8,6) | 3.7 (2,5.4) | 3.2 (1.9,4.5) | 0.230 |
|  | [40, 60] | 21.3 (17.5,25.2) | 18.5 (15,21.9) | 23.7 (20.3,27.1) | 22.9 (19.1,26.6) | 22.0 (19.2,24.8) | 22.6 (17.8,27.3) | 22.5 (19.2,25.8) | 0.500 |
|  | 60+ | 48.5 (45,52.1) | 49.2 (46.2,52.2) | 50.3 (47.8,52.8) | 49.3 (46.9,51.7) | 47.6 (45.2,49.9) | 48.1 (45,51.1) | 49.7 (47.5,51.9) | 0.940 |
| Race / Ethnicity | Mexican American | 12.1 (9.9,14.3) | 9.7 (6.9,12.4) | 12.2 (7.6,16.8) | 11.8 (8.2,15.3) | 13.5 (10,17.1) | 15.0 (11.6,18.4) | 12.5 (8,17) | 1.000 |
|  | Other Hispanic | 13.6 (8,19.2) | 13.1 (9.6,16.6) | 20.6 (6.8,34.3) | 14.5 (6.4,22.6) | 15.9 (13.1,18.6) | 15.7 (10.4,21) | 18.3 (12.7,23.8) | 0.670 |
|  | Non-Hispanic White | 21.2 (17.8,24.5) | 20.3 (18.2,22.3) | 23.6 (20.9,26.3) | 23.7 (20.9,26.6) | 23 (21.1,25) | 23.7 (20.9,26.4) | 24.8 (21.5,28.1) | 0.810 |
|  | Non-Hispanic Black | 22.0(19.3,24.6) | 23.7 (19.8,27.7) | 25.1 (22.5,27.7) | 24.6 (20.8,28.4) | 27.6 (24.7,30.6) | 27.7 (22.9,32.4) | 27.2 (23.6,30.7) | 0.440 |
|  | Other | 17.9 (7.7,28.1) | 20.5 (10.7,30.4) | 19.3 (14.6,23.9) | 22.7 (16.1,29.2) | 17.7 (11.5,24) | 17.5 (12.9,22) | 17.0 (11.7,22.3) | 0.070 |
| Education level | < High school | 24.8 (21.1,28.5) | 25.3 (21.8,28.8) | 28.6 (22.8,34.4) | 26.6 (22.3,30.9) | 25.9 (22.5,29.3) | 26.0 (23.3,28.8) | 26.6 (21.6,31.6) | 0.950 |
|  | High school | 23.0 (19.2,26.7) | 21.7 (18.3,25.2) | 25.8 (22.2,29.4) | 25.8 (20.7,30.9) | 23.7 (21.3,26.1) | 24.9 (20.9,29) | 24.8 (19.6,30) | 0.070 |
|  | > High school | 16.1 (13.8,18.3) | 16.5 (14.1,18.9) | 19.0 (16.8,21.2) | 19.8 (17.1,22.5) | 19.9 (16.9,22.9) | 20.4 (17,23.8) | 21.7 (18.2,25.2) | 0.440 |
| Income to poverty ratio | ≤ 1.3 | 20.9 (17.5,24.3) | 19.0 (16.6,21.4) | 22.4 (18.8,26.1) | 23.4 (18.9,27.9) | 21.1 (18.6,23.7) | 20.7 (17.9,23.4) | 21.7 (17.2,26.2) | 0.790 |
|  | 1.3–3.5 | 19.1 (16.2,22) | 20.8 (17.6,24) | 23.7 (20.6,26.9) | 24.4 (20.9,27.8) | 24.8 (22.5,27) | 24.6 (21.6,27.5) | 23.5 (21.4,25.6) | 0.920 |
|  | > 3.5 | 18.7 (14.7,22.6) | 17.9 (14.8,20.9) | 21.9 (18.4,25.4) | 20.3 (17.7,23) | 20.5 (18.1,22.9) | 21.9 (17.4,26.3) | 23.1 (19.3,26.9) | 0.460 |
| Marital status | Married/living with partner | 22.1 (19.3,25) | 18.9 (16.4,21.4) | 22.6 (20.6,24.6) | 21.0 (18.8,23.2) | 22.3 (20.5,24.2) | 23.2 (20.3,26.2) | 23.4 (20.3,26.5) | 0.760 |
|  | Other | 17.5 (14.2,20.8) | 20.6 (17.1,24.1) | 22.7 (19.3,26.2) | 25.5 (21,30) | 21.6 (18.9,24.3) | 21.2 (19,23.4) | 22.7 (19.3,26.1) | 0.800 |
| BMI (kg/m^2^) | < 25 | 11.0 (9,13.1) | 12.1 (9.5,14.7) | 12.8 (10.3,15.2) | 13.7 (11.4,16) | 14.8 (13,16.6) | 12.4 (10.1,14.8) | 14.4 (10.1,18.7) | 0.710 |
|  | 25–29 | 20.9 (16.9,24.9) | 19.5 (16.7,22.4) | 24.2 (21.1,27.3) | 23.5 (19.5,27.5) | 21.0 (17.3,24.7) | 22 (18.3,25.7) | 21.6 (19,24.2) | 0.130 |
|  | 30+ | 29.2 (25.7,32.7) | 25.9 (22.8,29) | 30.8 (27.6,34) | 29.8 (26.8,32.7) | 29.3 (26.8,31.7) | 31.5 (29.4,33.5) | 31.3 (27.5,35) | 0.430 |

Note: Data are presented as percentages (95% confidence interval), unless otherwise indicated. ^1^The overall *P*-value was adjusted for all characteristics and the *P*-value for each subgroup was adjusted for the rest of the characteristics.

**Supplementary Table 4**. Simultaneous treatment for hypertension and diabetes, hypercholesterolemia and diabetes, hypertension and hypercholesterolemia among adult participants (1999-2012) with these three conditions, respectively

|  | | Prevalence as percentage (95% confidence interval) | | | | | | | |  |
| --- | --- | --- | --- | --- | --- | --- | --- | --- | --- | --- |
| Combinations | | Hypertension and Diabetes | |  | Hypercholesterolemia and Diabetes | |  | Hypertension and Hypercholesterolemia | |  |
|  | | 1999–2006  (N =1,291) | 2007–2012  (N = 1,690) | *P*^1^ | 1999–2006  (N = 796) | 2007–2012  (N = 1,362) | *P* | 1999–2006  (N = 1,603) | 2007–2012  (N = 2,340) | *P* |
| Overall |  | 73.6 (70.3,77) | 79.2 (76.7,81.8) | 0.006 | 71.7 (67.7,75.7) | 79.0 (75.4,82.6) | 0.08 | 88.1 (85.9,90.3) | 90.7 (88.7,92.6) | 0.410 |
| Gender | Male | 69.7 (64.2,75.2) | 79 (74.9,83.2) | 0.006 | 70.2 (64.2,76.3) | 79.6 (75.4,83.7) | 0.04 | 88.9 (85.8,92.1) | 91.5 (88.9,94) | 0.780 |
|  | Female | 76.8 (72.4,81.2) | 79.5 (76.5,82.4) | 0.41 | 73.2 (66.8,79.5) | 78.4 (72.6,84.3) | 0.56 | 87.3 (84.4,90.1) | 90 (87.2,92.7) | 0.220 |
| Age (years) | [20, 40] | 66.5 (49.1,83.9) | 76.6 (62.9,90.3) | 0.99 | 78.9 (54,103.8) | 74.2 (59.4,88.9) | 0.56 | 74.2 (56.1,92.3) | 87.2 (75.7,98.7) | 0.240 |
|  | [40, 60] | 71.5 (66.5,76.4) | 77.3 (72.8,81.8) | 0.04 | 66.5 (58.9,74) | 76.3 (70.4,82.1) | 0.14 | 85.3 (81,89.5) | 88.2 (84.5,91.8) | 0.780 |
|  | 60+ | 75.5 (70.8,80.2) | 80.6 (77.9,83.3) | 0.12 | 75.0 (69.9,80.1) | 81.1 (77.3,84.8) | 0.14 | 90.4 (88.2,92.7) | 92.2 (90.2,94.3) | 0.180 |
| Race | Mexican American | 80.1 (74.2,85.9) | 83.0 (78.6,87.3) | 0.74 | 79.0 (73,85.1) | 77.5 (72.1,83) | 0.53 | 89.3 (84.7,93.8) | 93.9 (88.3,99.4) | 0.550 |
|  | Other Hispanic | 66.9 (52,81.8) | 77.4 (72.2,82.6) | 0.70 | 60.2 (40.3,80.1) | 70.1 (61.1,79.2) | 0.38 | 80.3 (65.6,94.9) | 86 (81.5,90.6) | 0.580 |
|  | Non-Hispanic White | 72.6 (67.8,77.5) | 79.7 (75.5,83.9) | 0.01 | 70.3 (65.3,75.4) | 81.5 (76.8,86.2) | 0.01 | 87.9 (85.2,90.7) | 90.7 (88.6,92.9) | 0.330 |
|  | Non-Hispanic Black | 76.4 (71.5,81.4) | 76.7 (72.7,80.7) | 1.00 | 78.6 (70.5,86.7) | 77.6 (73.2,82) | 0.42 | 91.5 (88.3,94.8) | 92.4 (90.1,94.8) | 0.660 |
|  | Other | 73.8 (60.5,87) | 80.0 (69.3,90.6) | 0.93 | 79.2 (66.1,92.3) | 70.8 (55.3,86.2) | 0.33 | 87.2 (76.4,97.9) | 86.9 (77.7,96.1) | 0.880 |
| Education | < High school | 78 (73.4,82.5) | 79.2 (76.1,82.3) | 0.94 | 77.2 (71.5,83) | 77.9 (73.2,82.6) | 0.81 | 87.6 (84.4,90.9) | 93.8 (91.1,96.4) | 0.020 |
|  | High school | 68.8 (62.3,75.2) | 78 (72.8,83.2) | 0.01 | 70.0 (63,76.9) | 77.3 (70.4,84.2) | 0.44 | 89.0 (84.2,93.9) | 89.9 (86.3,93.4) | 0.730 |
|  | > High school | 73.5 (68.6,78.4) | 79.9 (75.6,84.3) | 0.11 | 69 .0(62.6,75.4) | 80.6 (75.2,86) | 0.04 | 87.6 (84.3,90.9) | 89.8 (86.7,92.8) | 0.500 |
| Income to poverty ratio | ≤ 1.3 | 74.6 (68.7,80.6) | 79.5 (75.7,83.4) | 0.35 | 78.3 (69.3,87.4) | 78.4 (72.6,84.1) | 0.74 | 89.7 (85.9,93.5) | 91.8 (89.6,93.9) | 0.330 |
|  | 1.3–3.5 | 73.8 (68.6,78.9) | 78.8 (74.3,83.2) | 0.12 | 71.1 (63.7,78.4) | 76.7 (71,82.4) | 0.38 | 88.9 (85.4,92.5) | 90.1 (86.6,93.6) | 0.660 |
|  | > 3.5 | 70.8 (63.7,78) | 78.7 (73,84.5) | 0.19 | 67.1 (58.3,76) | 80.1 (73.7,86.5) | 0.04 | 87.8 (84,91.6) | 91.1 (87.5,94.6) | 0.570 |
| Marital status | Married/living with partner | 72.7 (67.6,77.9) | 80.1 (76.5,83.7) | 0.03 | 71.4 (66.8,76) | 78.4 (73.5,83.3) | 0.24 | 88.7 (85.8,91.5) | 91.4 (88.9,93.8) | 0.670 |
|  | Other | 74.2 (69.7,78.8) | 77.8 (74.1,81.5) | 0.32 | 72.4 (64.7,80.1) | 79.8 (74.2,85.3) | 0.24 | 87.5 (83.9,91) | 89.4 (86.7,92) | 0.320 |
| BMI (kg/m^2^) | < 25 | 76.7 (68.3,85.2) | 76.1 (67.5,84.8) | 0.64 | 74.7 (62.9,86.5) | 73.5 (64.2,82.9) | 0.19 | 89 (84.5,93.4) | 88.3 (83,93.7) | 0.990 |
|  | 25–29 | 69.7 (62.9,76.5) | 78.4 (73.3,83.5) | 0.08 | 70.9 (62.2,79.6) | 72.9 (66.7,79.1) | 0.68 | 88.2 (84.6,91.8) | 88.5 (84.8,92.3) | 0.490 |
|  | 30+ | 74.2 (70.7,77.6) | 79.7 (76.5,82.9) | 0.01 | 72.3 (67.5,77.2) | 81.4 (77.4,85.5) | 0.006 | 88.4 (85.4,91.4) | 92.3 (90,94.6) | 0.080 |

Note: Data are presented as percentages (95% confidence interval), unless otherwise indicated. ^1^*P*-values for comparisons were adjusted according to the characteristics of the participants.

**Supplementary Table 5.** Simultaneous control for hypertension and diabetes, hypercholesterolemia and diabetes, hypertension and hypercholesterolemia among adult participants (1999-2012) with these three combinations, respectively

|  | | Prevalence as percentage (95% confidence interval) | | | | | | | |  |
| --- | --- | --- | --- | --- | --- | --- | --- | --- | --- | --- |
| Combinations | | Hypertension and Diabetes | |  | Hypercholesterolemia and Diabetes | |  | Hypertension and Hypercholesterolemia | |  |
|  | | 1999–2006  (N = 1,399) | 2007–2012  (N = 1,733) | *P*^1^ | 1999–2006  (N = 1,423) | 2007–2012  (N = 1,754) | *P* | 1999–2006  (N = 4,051) | 2007–2012  (N = 3,868) | *P* |
| Overall |  | 16.4 (13.8,19) | 20.1 (16.4,23.8) | 0.14 | 9.1 (7,11.1) | 17.0 (14,20) | <.001 | 12.0(10.3,13.8) | 24.5 (22.4,26.6) | < 0.001 |
| Gender | Male | 16.9 (12.9,20.9) | 19.3 (14.1,24.5) | 0.59 | 10.3 (6.8,13.7) | 19.0 (15.4,22.7) | 0.01 | 16.6 (14,19.2) | 29.5 (27,32.1) | < 0.001 |
|  | Female | 16 (12.3,19.7) | 20.9 (16.8,24.9) | 0.12 | 7.9 (5.2,10.7) | 15.0 (10.7,19.4) | 0.02 | 8.4 (6.8,10) | 20.2 (17.4,22.9) | < 0.001 |
| Age (years) | [20, 40) | 10 (-0.7,20.6) | 33.9 (16.3,51.5) | 0.36 | 0.7 (-0.8,2.2) | 11,0 (2.2,19.8) | 0.08 | 2.0 (-0.1,4.2) | 10.7 (3.8,17.5) | < 0.001 |
|  | [40, 60) | 16.8 (11.9,21.6) | 21.9 (16.2,27.5) | 0.22 | 7.2 (3.9,10.4) | 15.3 (10.9,19.7) | 0.006 | 11.0 (8.5,13.6) | 21.3 (18.2,24.5) | < 0.001 |
|  | 60+ | 16.7 (13.4,20.1) | 18.2 (14.7,21.8) | 0.64 | 12.0 (9.2,14.9) | 19.2 (15.8,22.7) | 0.02 | 14.1 (11.8,16.4) | 28.3 (25.6,30.9) | < 0.001 |
| Race | Mexican American | 5.4 (2.9,7.8) | 11.9 (8.3,15.5) | 0.02 | 2.8 (1.4,4.2) | 10.4 (6.3,14.6) | <.001 | 8.0 (5.3,10.8) | 16.1 (13.3,19) | 0.020 |
|  | Other Hispanic | 7.4 (0.5,14.3) | 15.9 (10.4,21.3) | 0.22 | 8.6 (1.9,15.2) | 11.1 (7.2,15) | 0.38 | 6.5 (1,12) | 20.5 (14.2,26.7) | 0.054 |
|  | Non-Hispanic White | 19.3 (15.9,22.7) | 22.7 (17.1,28.3) | 0.35 | 10.9 (7.9,13.9) | 20,0 (16,24) | 0.005 | 12.9 (10.9,14.9) | 25.5 (23.2,27.8) | < 0.001 |
|  | Non-Hispanic Black | 12.1 (9,15.1) | 18.4 (15,21.9) | 0.02 | 5.6 (3.3,7.9) | 13.7 (10,17.5) | 0.002 | 10.6 (8,13.2) | 21.2 (18.5,23.9) | < 0.001 |
|  | Other | 18.3 (5.3,31.4) | 14 (4.9,23.1) | 0.10 | 6.9 (0.4,13.4) | 14.5 (4.5,24.4) | 0.45 | 9.7 (4.5,14.9) | 29.7 (20,39.5) | 0.001 |
| Education | < High school | 12 (8.8,15.1) | 16.9 (13.5,20.3) | 0.01 | 4.6 (3,6.3) | 13.2 (9.7,16.8) | <.001 | 11.1 (8.3,13.9) | 25.2 (21.2,29.1) | < 0.001 |
|  | High school | 18.2 (12.3,24.1) | 18.3 (12.5,24.2) | 0.75 | 11.2 (7.6,14.8) | 18.1 (12.5,23.8) | 0.06 | 14.2 (11.4,17) | 22.7 (19.6,25.8) | 0.001 |
|  | > High school | 19 (14.1,23.9) | 23.2 (17,29.4) | 0.38 | 11.1 (7.1,15.2) | 18.8 (14.4,23.1) | 0.06 | 11.2 (9.3,13.2) | 25.1 (22,28.2) | < 0.001 |
| Income to poverty ratio | ≤ 1.3 | 12.7 (8.5,17) | 16.3 (12.2,20.5) | 0.09 | 7.1 (4.4,9.8) | 11.3 (8.8,13.7) | 0.09 | 9.1 (6.5,11.8) | 22.7 (19.5,25.9) | < 0.001 |
|  | 1.3-3.5 | 14.7 (10.6,18.8) | 22.7 (16.8,28.5) | 0.09 | 8.2 (5.4,11.1) | 18.9 (13.1,24.7) | 0.001 | 11.6 (9.4,13.8) | 26.3 (23.5,29.1) | < 0.001 |
|  | > 3.5 | 22.2 (16.2,28.1) | 20.6 (13.6,27.5) | 0.70 | 13.0 (8.1,17.8) | 20.0 (13.9,26) | 0.09 | 14.6 (11.7,17.5) | 24.9 (21.4,28.4) | < 0.001 |
| Marital status | Married/living with partner | 18.0 (14.1,21.9) | 21.7 (17.4,25.9) | 0.36 | 10.3 (7.1,13.5) | 19.1 (15.5,22.7) | 0.005 | 13.7 (11.5,15.8) | 26.5 (23.5,29.4) | < 0.001 |
|  | Other | 15.1 (11.1,19) | 17.9 (13.2,22.6) | 0.23 | 7.7 (5.4,10.1) | 13.8 (10,17.5) | 0.02 | 9.6 (7.7,11.5) | 20.7 (18.1,23.2) | < 0.001 |
| BMI (kg/m^2^) | < 25 | 15.1 (8,22.3) | 16.3 (8.2,24.4) | 0.29 | 9.7 (5.9,13.5) | 22.1 (11.9,32.4) | 0.003 | 7.7 (5.4,10) | 15.0 (10.8,19.1) | 0.005 |
|  | 25–29 | 12.7 (9,16.4) | 19.2 (13.8,24.5) | 0.02 | 7.6 (3.9,11.3) | 18.5 (13.3,23.8) | 0.001 | 12.8 (10.2,15.4) | 22.1 (19,25.2) | < 0.001 |
|  | 30+ | 18.8 (14.9,22.6) | 20.9 (16.4,25.5) | 0.43 | 9.8 (6.7,13) | 15.4 (12.5,18.4) | 0.07 | 13.7 (11.3,16.1) | 29.4 (26.2,32.7) | < 0.001 |

Note: Data are presented as percentages (95% confidence interval), unless otherwise indicated. ^1^*P*-values for comparisons were adjusted for participants’ characteristics.
